# Supplementary material for: Global record-shattering breadbasket droughts emerge from moderately extreme regional events
Source: Nat Commun. 2026 Mar 17;17:2577. doi: 10.1038/s41467-026-70700-z (PMC13000154; doi:10.1038/s41467-026-70700-z)
Supplement: Supplementary file 1 — Supplementary Information [file 41467_2026_70700_MOESM1_ESM.pdf]

Supplementary Information for Global record-shattering breadbasket droughts emerge from moderately extreme regional events

Ji Li[1,*], Jakob Zscheischler[1,2], and Emanuele Bevacqua[1,*]

[1]Department of Compound Environmental Risks, Helmholtz Centre for Environmental Research - UFZ, Leipzig, Germany

[2]Department of Hydro Sciences, TUD Dresden University of Technology, Dresden, Germany

[*]Corresponding author: Ji Li (Email address: j.li@ufz.de), Emanuele Bevacqua (Email address: emanuele.bevacqua@ufz.de)

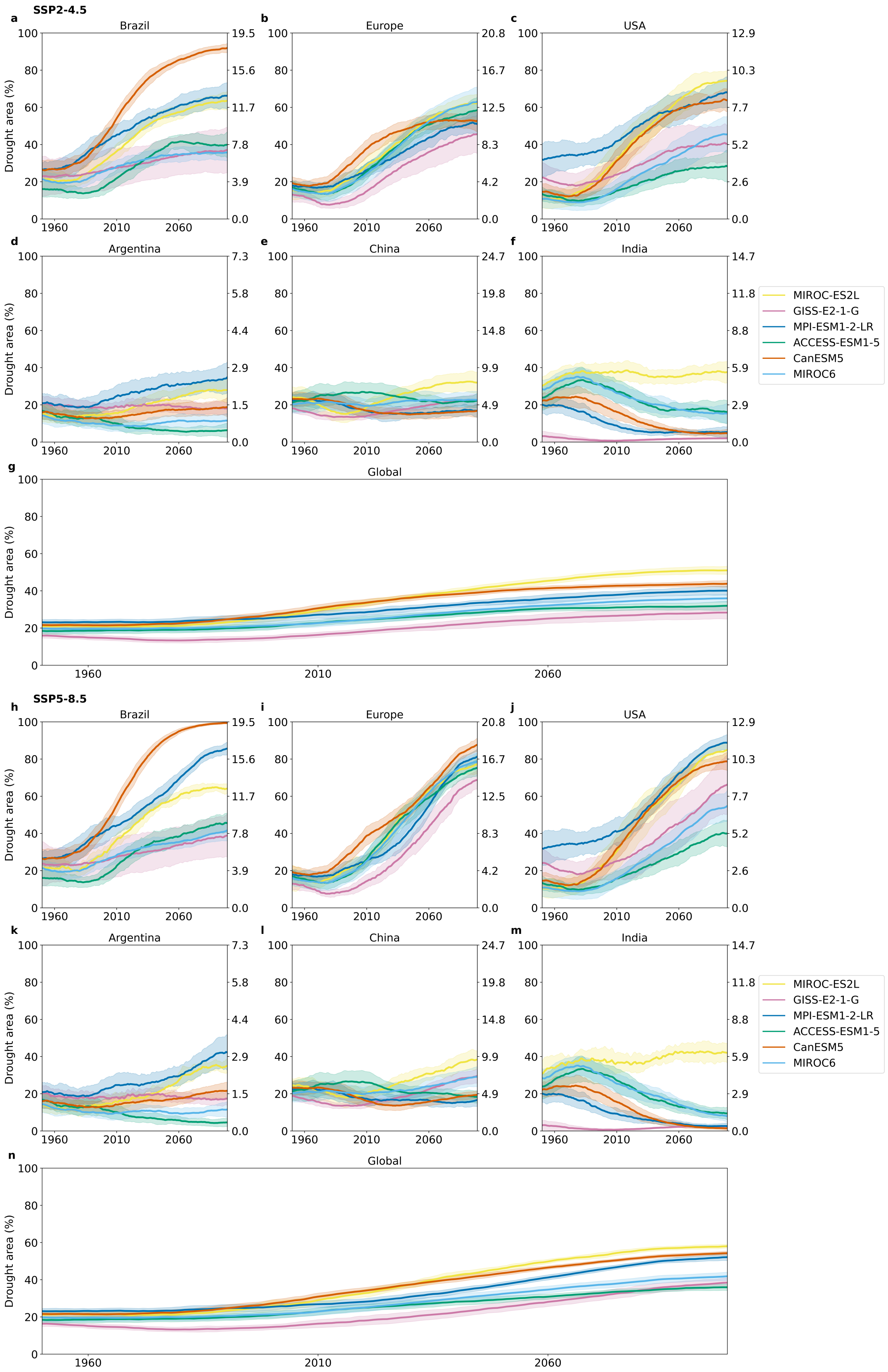

Supplementary Figure 1: **Annual global and regional drought areas. a-f**, Annual regional drought area for six major maize-producing regions under SSP2-4.5. The left y-axis shows the drought area relative to the respective regional area, while the right y-axis shows the same drought area expressed as a fraction of the global maize breadbasket area. **g**, Annual global drought area under SSP2-4.5. Solid lines represent the multi-ensemble mean for each model, and the shaded areas indicate the inter-member spread, shown as ±1 standard deviation across ensemble members. **h-n**, As panels **a-g**, but for SSP5-8.5.

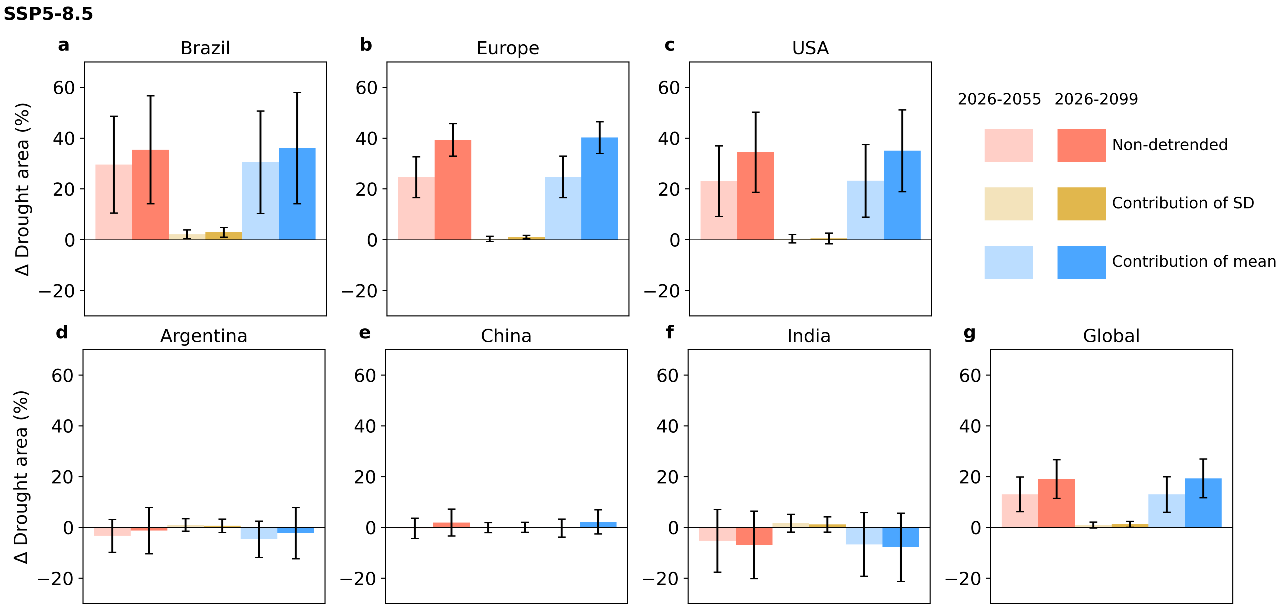

Supplementary Figure 2: **Projected changes in annual global and regional drought areas.** As Figure 2, but for SSP5-8.5.

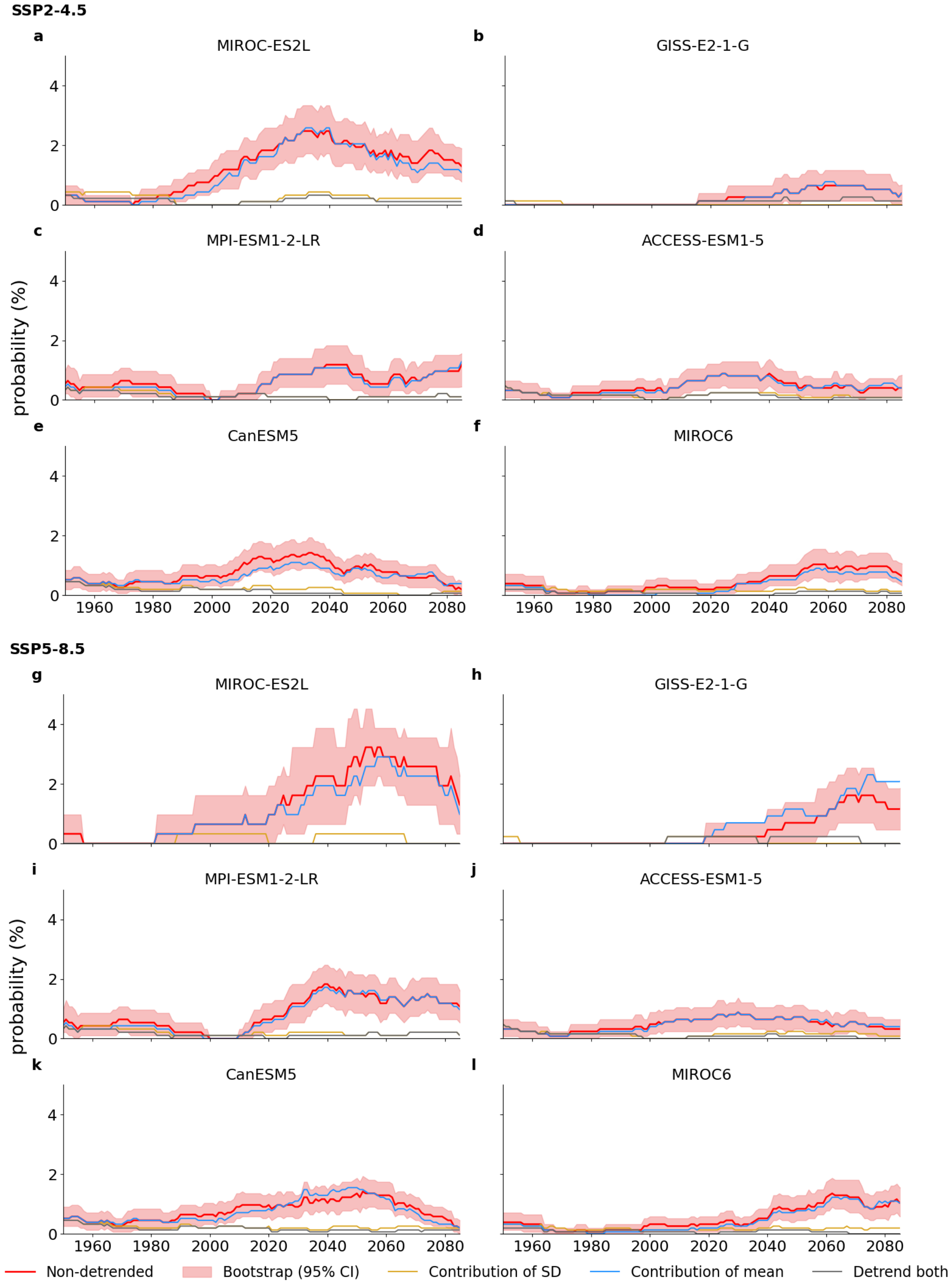

Supplementary Figure 3: **Annual probability of global record-shattering droughts.** **a-f**, Probability under SSP2-4.5 based on different models (stated in the title of the panels). The red line shows the probability based on the original (non-detrended) data (consistent with other record-shattering probabilities in the paper, based on pooled ensemble members of each model). For each model, the shading shows the centered 95% confidence intervals derived via bootstrap (1000 resamples of the ensemble members of the model) to quantify the uncertainty from internal variability. Other lines show the contribution to the probabilities from changes in surface soil moisture's standard deviation (SD, yellow), mean (blue), and both mean and standard deviation (grey) (Methods). **g-l**, As panels **a-f**, but for SSP5-8.5.

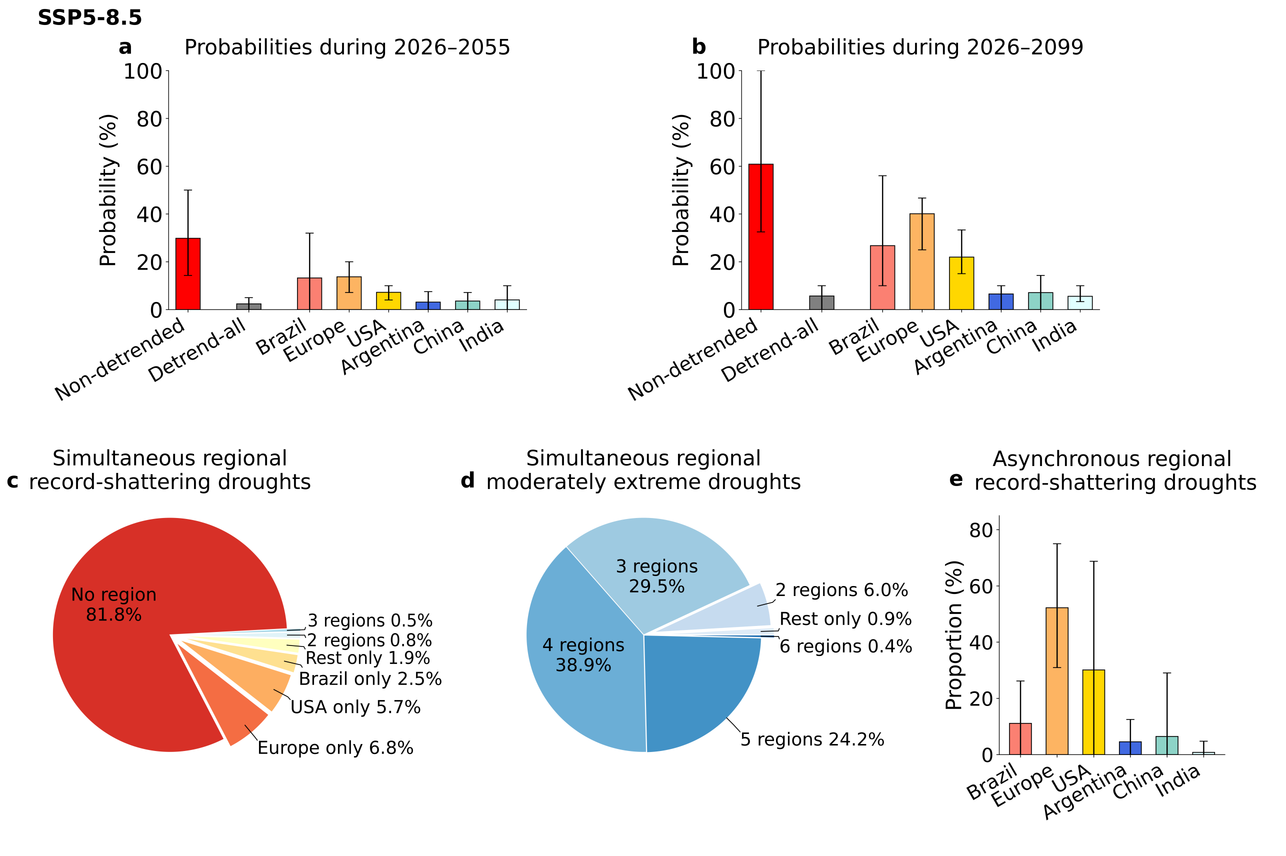

Supplementary Figure 4: **Contributions of regional surface soil moisture trends and timing of regional droughts relative to global events.** As Figure 4, but for SSP5-8.5.

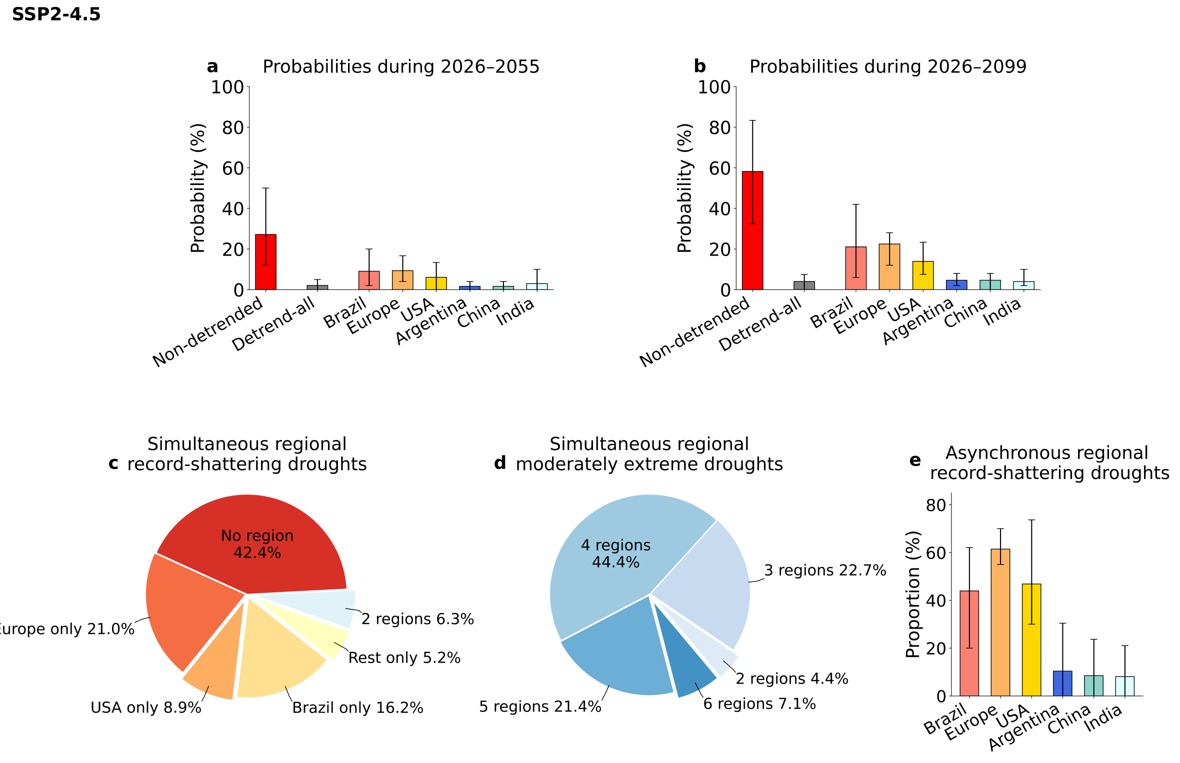

Supplementary Figure 5: **Contributions of regional surface soil moisture trends and timing of regional droughts relative to global events.** As Figure 4, but for drought defined with a 5th percentile threshold.

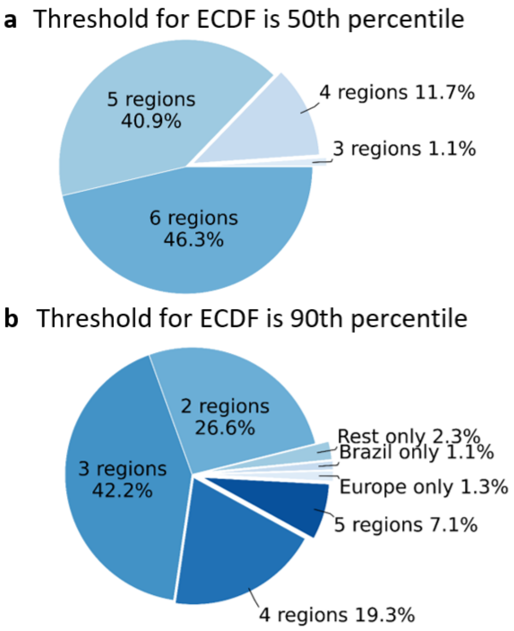

Supplementary Figure 6: **Sensitivity of the simultaneous regional drought patterns to the definition of moderately extreme droughts under SSP2-4.5.** As Figure 4**d**, but for moderate drought defined using the 50th percentile threshold (**a**) and 90th percentile threshold (**b**).

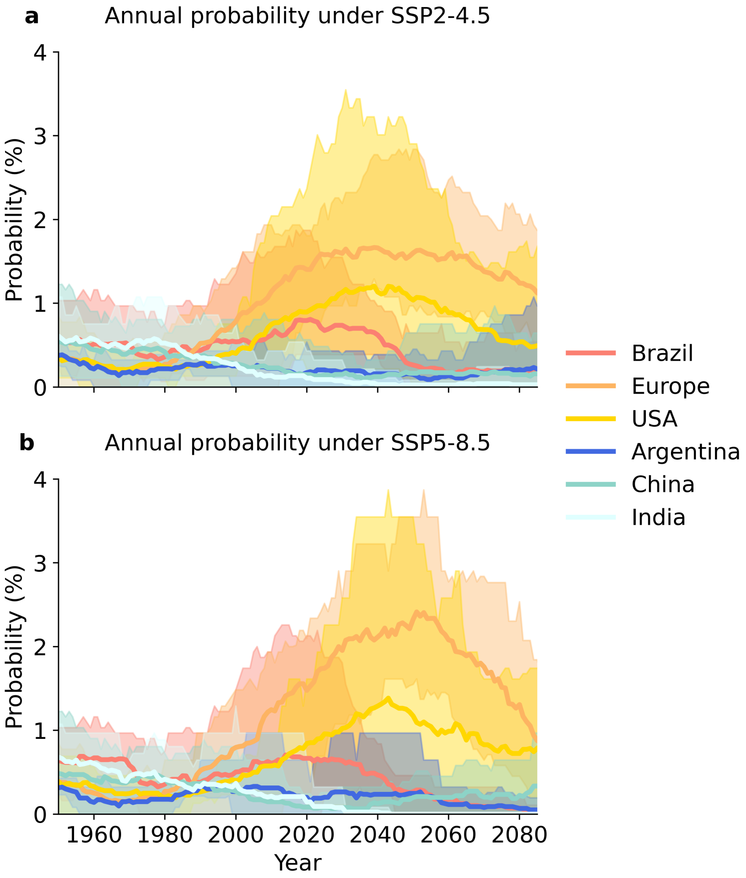

Supplementary Figure 7: **Annual probability of regional record-shattering droughts.** **a**, Probability under SSP2-4.5, where solid lines show the multi-model mean for each region, and shading shows the inter-model range. **b**, As panel **a**, but for SSP5-8.5

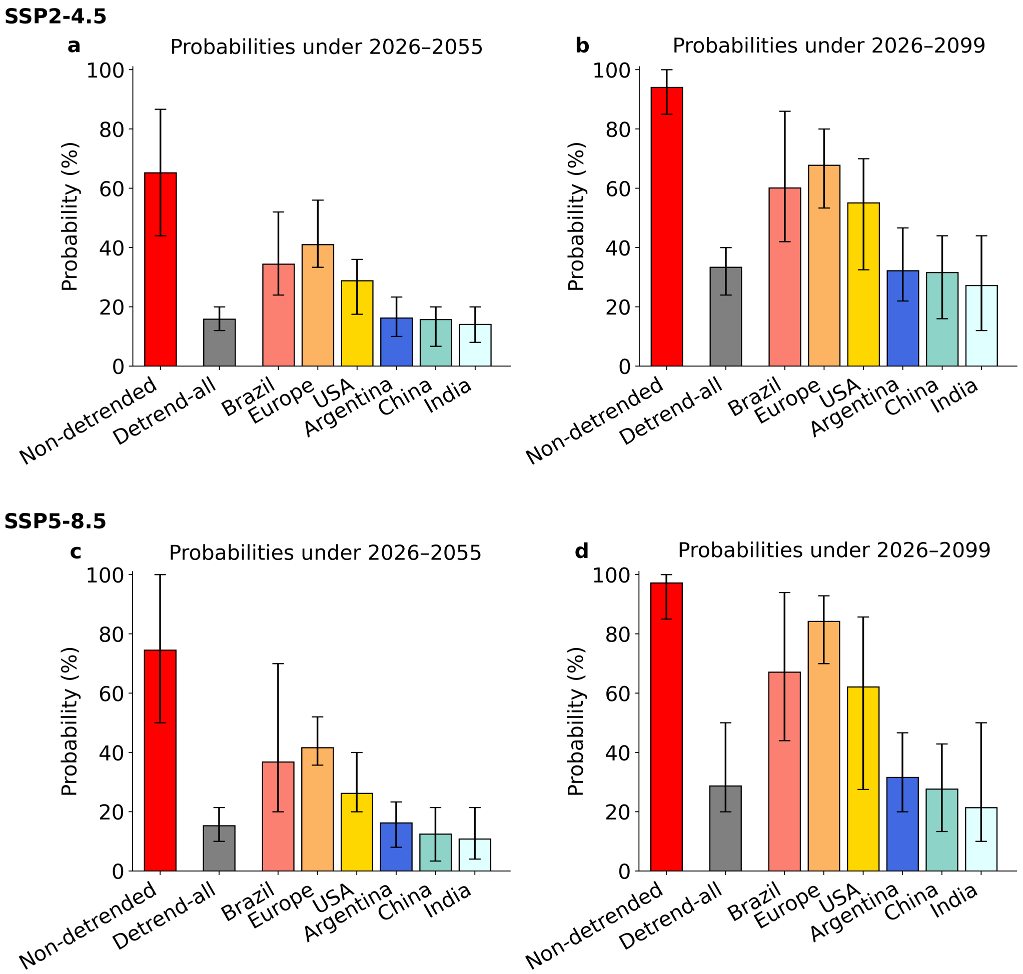

Supplementary Figure 8: **Contributions of regional surface soil moisture trends to global record-breaking droughts.** As Figure 4**a,b**, but here **a,b** show the probability of having at least one global record-breaking (instead of record-shattering) drought under SSP2-4.5 during 2026-2055 (**a**) and 2026-2099 (**b**). **c,d**, As panels **a,b**, but under SSP5-8.5.

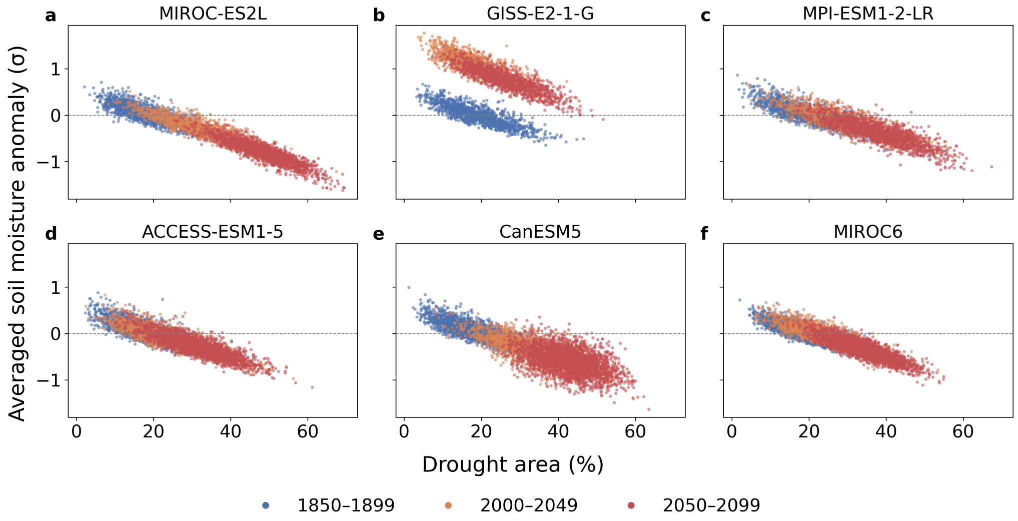

Supplementary Figure 9: **Association between drought spatial extent and mean drought intensity. a-f**, Scatter plot of annual global drought area versus the area-weighted mean of standardized surface soil moisture anomalies across all grid cells under drought, for individual models. Each point represents the annual value from one ensemble member. Colors denote three time periods (1850-1899, 2000-2049, and 2050-2099).

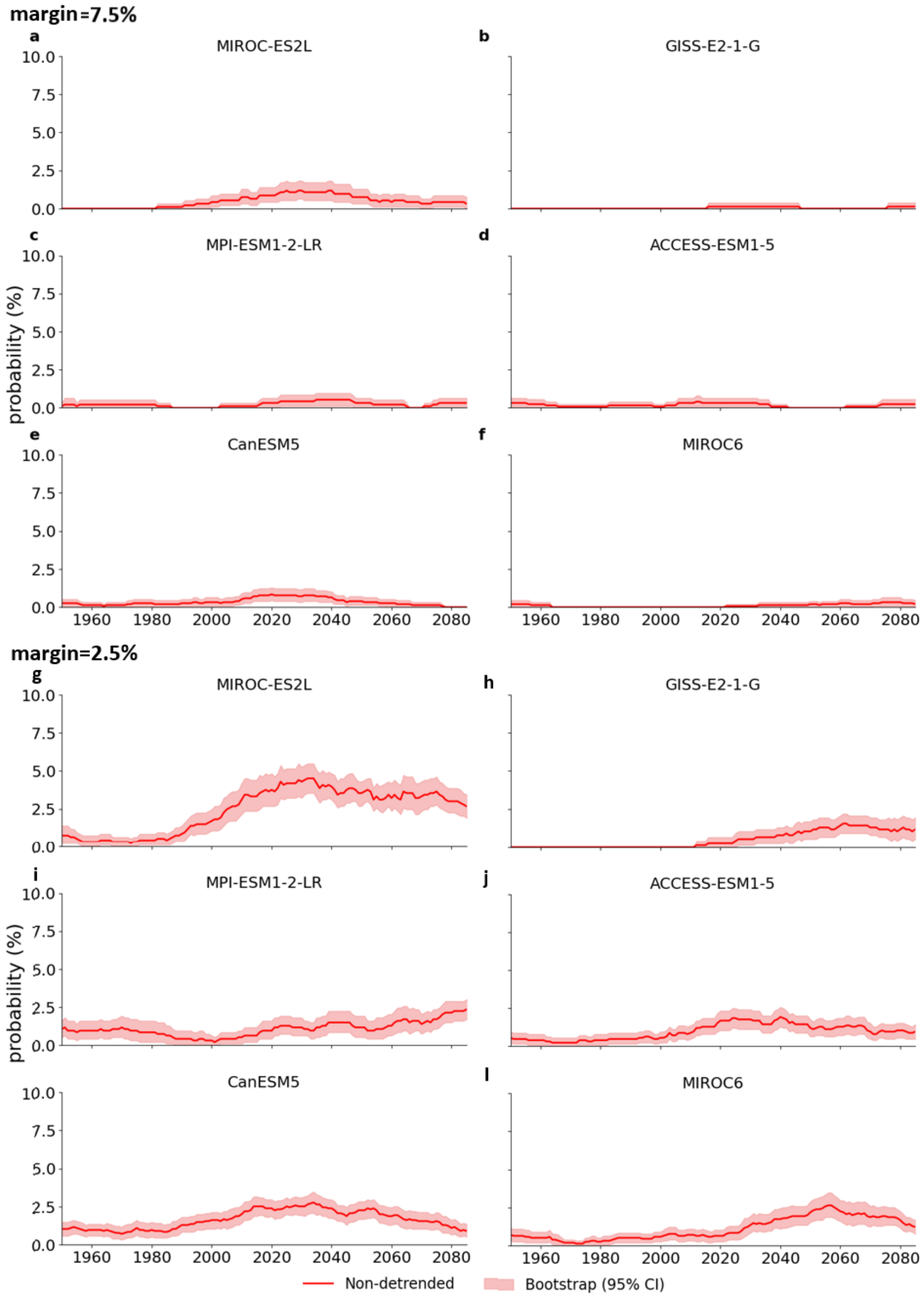

Supplementary Figure 10: **Annual probability of global record-shattering droughts under SSP2-4.5.** As Supplementary Figure 3, but here **a-f** show the probability based on the original (non-detrended) data and the centered 95% confidence intervals derived via bootstrap calculated with a margin of 7.5% to define the record-shattering drought. **g-l**, same as **a-g**, but used the margin of 2.5%.

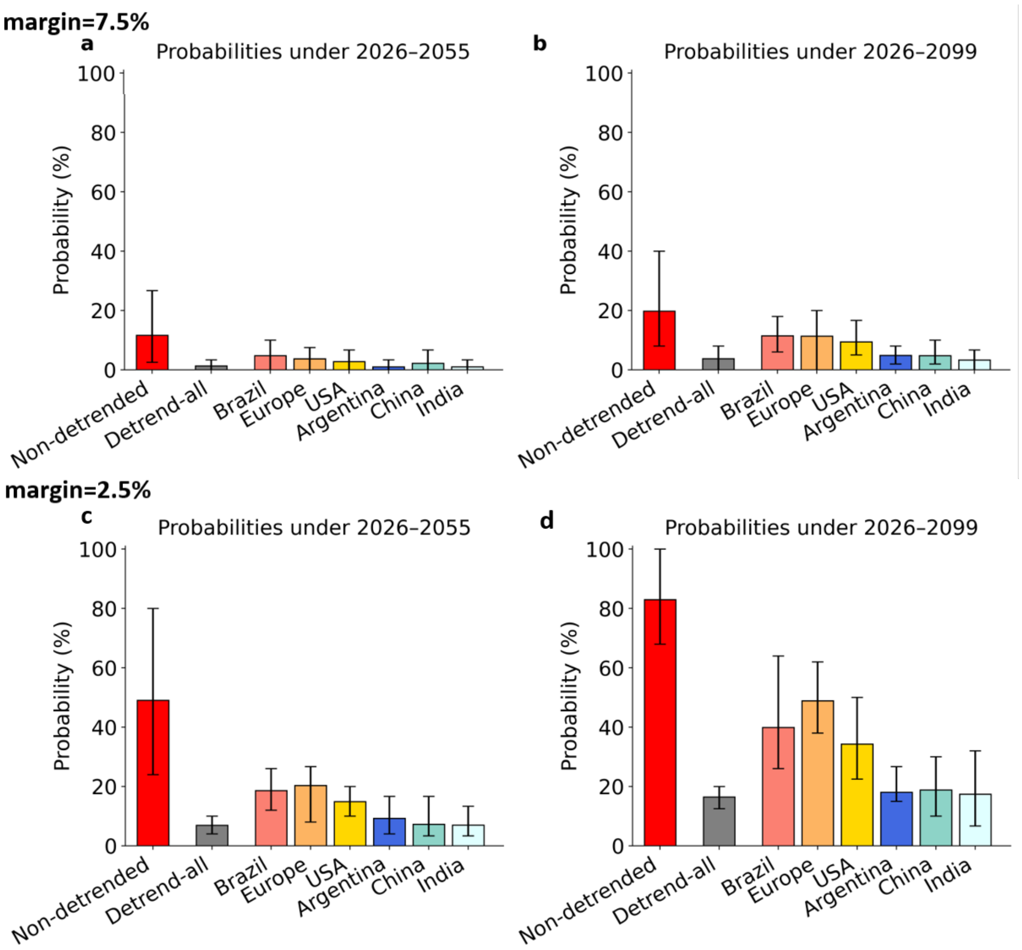

Supplementary Figure 11: **Contributions of regional surface soil moisture trends to global record-shattering droughts identified by different margins.** As Figure 4**a,b**, but here **a,b** show the probability of having at least one global record-shattering drought with a 7.5% margin under SSP2-4.5 during 2026-2055 (**a**) and 2026-2099 (**b**). **c,d**, As panels **a,b**, but calculated with a 2.5% margin.

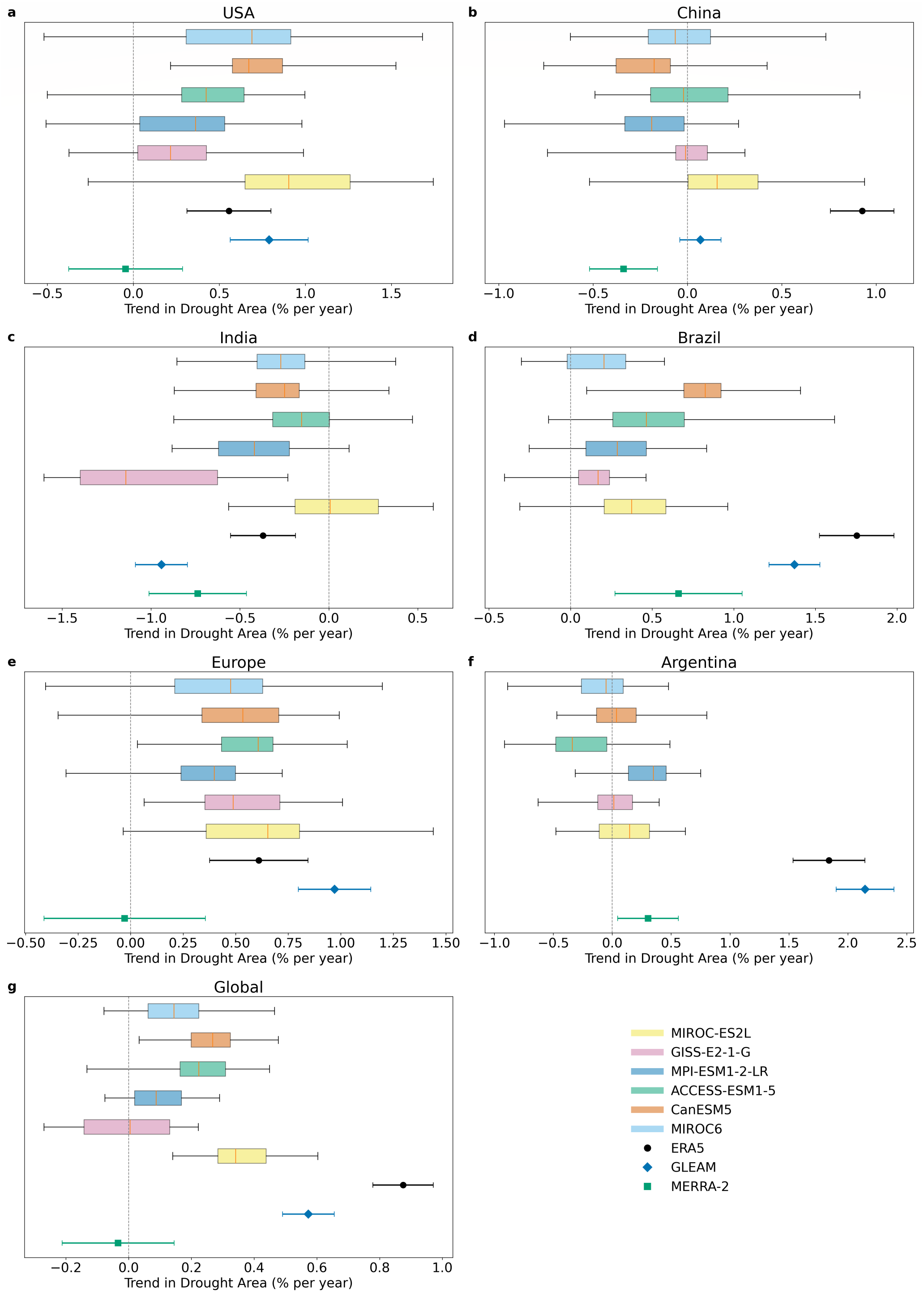

Supplementary Figure 12: **Regional and global drought area trends in observation based datasets and SMILEs under SSP2-4.5**. **a-g**, the distribution of linear trends in drought area (%) across ensemble members of individual SMILEs models for each region and globally. Observed drought area time series derived from ERA5, GLEAM, and MERRA-2 are shown as thick black, blue, and green lines, respectively. Error bars represent uncertainty in the observational trend, quantified as the standard deviation of the linear regression slope fitted to annual drought area time series over 1980-2024.

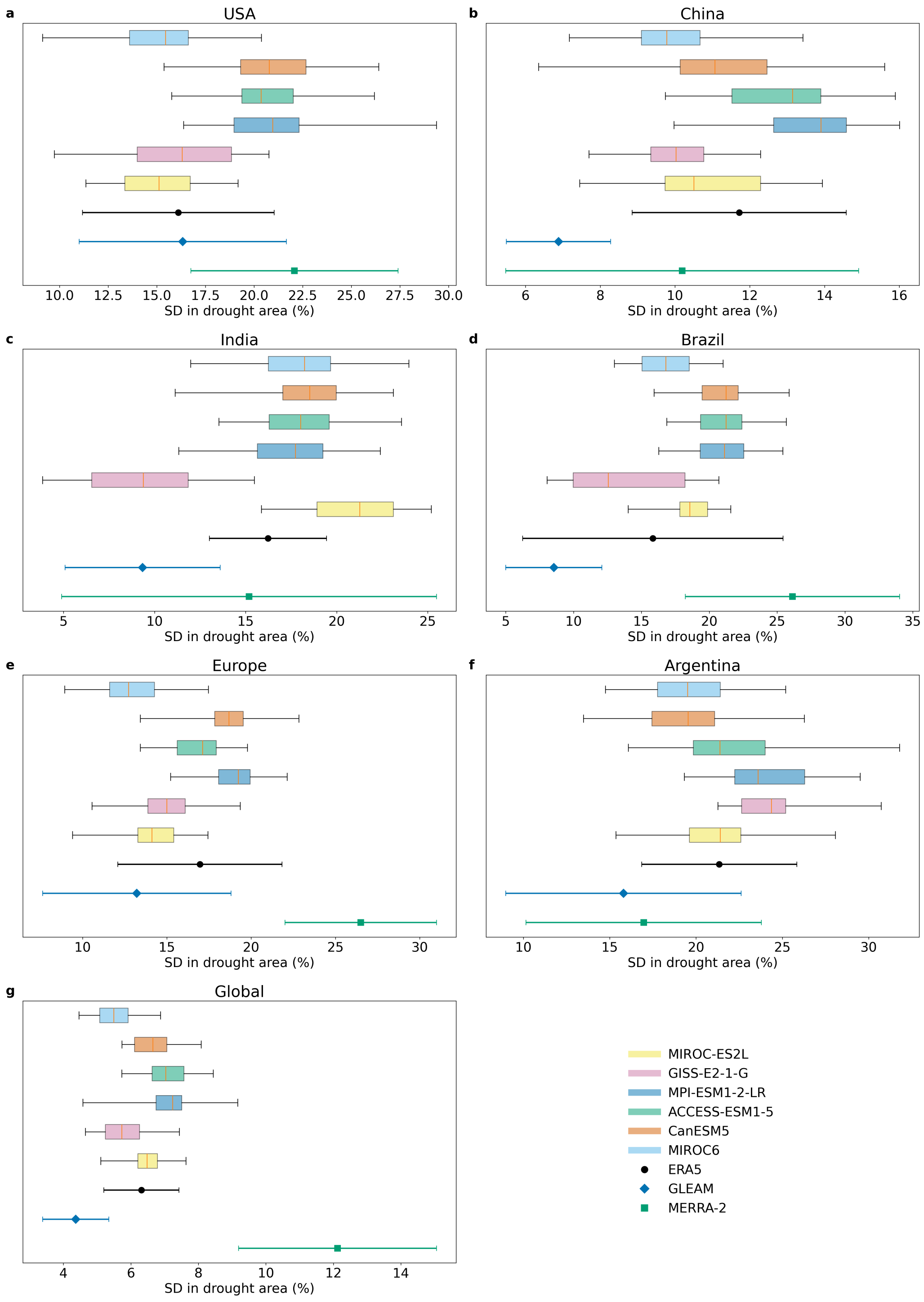

Supplementary Figure 13: **Regional and global drought area standard deviation (SD) in observation based datasets and SMILEs under SSP2-4.5**. Same as Supplementary Figure 12 but for the distribution of the standard deviation (SD) of annual drought area (%).Observational estimates derived from ERA5, GLEAM, and MERRA-2 are shown as black circles, blue diamonds, and green squares, respectively.
